# Preparation of sisal fiber/polyaniline/bio-surfactant rhamnolipid-layered double hydroxide nanocomposite for water decolorization: kinetic, equilibrium, and thermodynamic studies

**DOI:** 10.1038/s41598-023-38511-0

**Published:** 2023-07-13

**Authors:** Mehrdad Negarestani, Soheil Tavassoli, Saba Reisi, Negar Beigi, Afsaneh Mollahosseini, Majid Hosseinzadeh, Asiyeh Kheradmand

**Affiliations:** 1 Department of Civil and Environmental Engineering, Iran University of Science and Technology (IUST), Narmak, Tehran, Iran; 2 Research Laboratory of Spectroscopy & Micro and Nano Extraction, Department of Chemistry, Iran University of Science and Technology (IUST), Narmak, Tehran, Iran; 3 Department of Environmental Engineering, Graduate Faculty of Environment, University of Tehran, Tehran, Iran

**Keywords:** Chemical engineering, Civil engineering

## Abstract

Sisal fiber is a potent economical biomaterial for designing composites because of its low density, high specific strength, no toxic effects, and renewability. The present study utilized sisal fiber as a starting material and subjected it to modification to produce a sisal fiber/polyaniline/bio-surfactant rhamnolipid-layered double hydroxide nanocomposite material denoted as SF@PANI@LDH@RL. The composite was evaluated for its efficacy in removing reactive orange 16 (RO16) and methylene blue (MB) from aqueous solutions. The synthesized adsorbent was characterized by FTIR, XRD, and SEM–EDS techniques; these analyses indicated the successful modification of the sisal fiber. The primary factors, including contact time, adsorbent dosage, dye concentration, temperature, and pH, were optimized for achieving the most excellent adsorption efficiency. On the one hand, methylene blue removal is enhanced in the basic solution (pH = 10). On the other hand, reactive orange 16 adsorption was favored in the acidic solution (pH = 3). The highest adsorption capacities for methylene blue and reactive orange 16 were 24.813 and 23.981 mg/g at 318 K, respectively. The Temkin isotherm model, which proves the adsorption procedure of methylene blue and reactive orange 16 could be regarded as a chemisorption procedure, supplies the most suitable explanation for the adsorption of methylene blue (R^2^ = 0.983) and reactive orange 16 (R^2^ = 0.996). Furthermore, Elovich is the best-fitting kinetic model for both dyes (R^2^ = 0.986 for MB and R^2^ = 0.987 for RO16). The recommended SF@PANI@LDH@RL adsorbent was reused six consecutive times and showed stable adsorption performance. The results demonstrate that SF@PANI@LDH@RL is a perfect adsorbent for eliminating cationic and anionic organic dyes from aqueous media.

## Introduction

Due to the dismissal of contaminants into different habitats, eliminating industrial pollutants, such as plastic, refineries, petroleum, printing, textile, and leather, has become one of the most significant universal problems^1–6^. In general, dyes are one of the most harmful materials in industrial effluents. The utilization of dyes has been found to cause significant and irreversible damage to the environment, posing a serious threat to both marine species and humans. This is primarily due to the slow decomposition and inherent toxicity of these substances. Based on the functional groups, textile dyes are categorized. Functional groups such as indigo, sulfur, nitro, nitroso, anthraquinone, and azo. Textile dyes are toxic, resistant, bioaccumulative, non-biodegradable, and carcinogenic and cause damaging consequences on the surroundings, even at low concentrations^7–12^. Classifying dyes based on the remaining particle charge after dissolution in an aqueous solution is standard. Such classifications consist of cationic (all basic dyes) and anionic (acidic, direct, and reactive dyes), and non-ionic (dispersed dyes)^13,14^. Reactive Orange 16, a xenobiotic monoazo dye and highly water-soluble recalcitrant (pka = 3.75), is basically dangerous and has carcinogenic and mutagenic impacts on people. Methylene blue (Methylthioninium chloride), a subset of the thiazine family, is a non-biodegradable, water-soluble cationic dye with a pka of 3.8. Therefore, the removal of dyes from the effluent is assumed to be an environmental issue^14–17^. Numerous methodologies have been employed for the elimination of synthetic dyes from polluted water. These include techniques such as extraction, membrane separation, adsorption, photocatalytic degradation, biological treatment, oxidation, filtration, flocculation, and coagulation^18–22^. Owing to being uneconomical, complex, and time-consuming, some of these standard techniques have been limited^23^. Discovering the most coherent and straightforward dye wastewater treatment approach is crucial. Adsorption is the best choice due to its clarity and flexibility in design and insensitivity to toxic contaminants in recent decades^24^. The quality of the adsorbent plays a crucial role in adsorption efficiency. Various types of sorbents, such as zeolite, silica, clay, chitosan, biochar, synthetic adsorbents (e.g. waste rubber tires, active carbon, mesoporous carbon substances, polymers, and filter membranes), have been studied for their effectiveness in removing pollutants from wastewater^25,26^. The layered double hydroxide (LDH), also known as the hydrotalcite-like layered structure, is a highly effective adsorbent that enables adjustable usage densities and enhances chemical homogeneity to a significant extent. The diversity of structural compositions, morphologies, and multiple synthetic strategies make LDHs readily engineered for specific adsorption processes with enhanced performance. The anionic interaction ability of LDH is significantly influenced by its design, which involves the incorporation of divalent and trivalent metal ions within the layers. This design is dependent on synthetic requirements and the intended application of the LDH^27^. The general formula for layered double hydroxide is shown as ${[{M}_{1-x}^{2+} {M}_{x}^{3+} {\left(OH\right)}_{2}]}^{x+} {{[A}^{n-}]}_{x/n}\cdot z{H}_{2}O$, where ${M}^{2+}$ and ${M}^{3+}$ are divalent cation (such as ${Co}^{2+}$, ${Ni}^{2+}$, ${Cu}^{2+}$, ${Zn}^{2+}$, ${Ca}^{2+}$, ${Mg}^{2+}$), and trivalent cation (such as ${Ga}^{3+}$, ${Al}^{3+}$, ${Fe}^{3+}$, ${Cr}^{3+}$), respectively. The typical anions representing the interlayer of layered double hydroxides are chloride, nitrate, hydroxide, carbonate, sulfate, and larger anion similar to polyoxometalates^28^. Because the anion with the negative charge assists in maintaining the brucite-like sheets with positive charge via electrostatic attractions, the entire charge of LDHs is positive. Different parts of these layered materials, such as low price and simple synthesis, high surface area, ion exchange ability, and swelling capabilities, make them a proper choice for a dye adsorbent^29^. Rhamnolipids (RL) are a category of anionic glycolipid biosurfactants created by different bacteria, such as Pseudomonas aeruginosa. The aforementioned substitutes for synthetic chemical surfactants in cosmetics and medicines exhibit potential due to their eco-friendliness and minimal toxicity. The molecular structure of rhamnolipids consists of 3-(3-hydroxyalkanoyloxy) alkanoate, wherein hydrophobic acyl chains are linked to a hydrophilic component comprising no more than two rhamnose molecules. An enhanced evaluation of biosurfactant utilization, particularly rhamnolipid-based ones, can be advantageous for the textile dye industry^30,31^. So far, only a limited number of studies have explored the effect of modified cellulusic fiber materials coupled with LDHs on emerging pollutant elimination. For instance, a polypyrrole/polyaniline modified composite based on sisal was synthesized for capturing dyes from water. The maximum adsorption capacity of 12.43 mg/g was reported for reactive orange 5^32^. In another study, cellulosic sisal fiber supported with conducting polymers was developed for the adsorption of ibuprofen from synthetic wastewater, with a maximum adsorption capacity of 21.33 mg/g^33^. So far, no study has been identified evaluating the synergic effects of rhamnolipid, LDH, and polyaniline as modifiers on sisal fiber for the removal of RO16 dye and methylene blue.

For the first time in this study, a potent platform was designed using SF@PANI@LDH to evaluate the removal efficiency of methylene blue and Reactive Orange 16 from effluent wastewater. The proposed adsorbent was characterized using FTIR, XRD, and SEM–EDS techniques. Different kinetic and isotherm models were employed to explain the adsorption process.

## Experimental

### Chemicals and equipment

Sisal fibers utilized in this study were purchased from a city bazaar in Tehran, Iran. Rhamnolipid (≥ 90%) and metal salts, including nickel nitrate (Ni(NO_3_)_2_0.6H_2_O), and aluminum nitrate (Al(NO_3_)_3_0.9H_2_O) were provided from sigma and Aldrich. All other chemicals like Aniline (≥ 99.5%), ammonium peroxydisulfate (APS), and urea were supplied from Merck, Germany, without any remediation. Hydrochloric acid and methanol were supplied from Merck (Germany) with 99% purity. Methylene blue (${\mathrm{C}}_{16}{\mathrm{H}}_{18}{\mathrm{CIN}}_{3}{\mathrm{Na}}_{3}\mathrm{S}$, 319.86 g/mol), and reactive orange 16 (${\mathrm{C}}_{20}{\mathrm{H}}_{17}{\mathrm{N}}_{3}{\mathrm{Na}}_{2}{\mathrm{O}}_{11}{\mathrm{S}}_{3}$, 617.32 g/mol) were purchased from Tamadkala Company (Iran). Deionized (DI) water was used in all the experimentations.

### Instruments and measurements

Modified sisal fiber morphology was examined using field emission scanning electron microscopy (SEM/ EDS, Iran). The chemical structure of the modified sisal fiber was analyzed by Fourier transform infrared spectroscopy (FTIR) (Thermo AVATAR IR spectrophotometer) in the spectral range of 4000–400 ${cm}^{-1}$. Powder X-ray diffraction (XRD) was exploited to analyze the physical quality of the materials. A Uv–vis spectrophotometer measured the concentrations of MB and RO16 during adsorption analyses.

### Synthesis of SF@PANI@LDH@RL composite

Distilled water and methanol were used to clean the sisal fibers thoroughly, and then the fibers were dried in the oven at 328 K. The polyaniline-modified sisal fiber (SF@PANI) was fabricated in 3 steps. Initially, the cleaned sisal fibers were added into the methanol aniline solution (v/v, 20:80) and soaked for 2 h. After that, the sisal fibers were removed from the methanol aniline solution and added to the HCL solution (0.175 M), which contained 8.75 $g/{L}^{-1}$ ammonium persulfate. Subsequently, it was stirred moderately at ambient temperature and pressure for 2 h. Finally, the sisal fibers were washed with water entirely and dried at 328 K.

**Figure 1:**
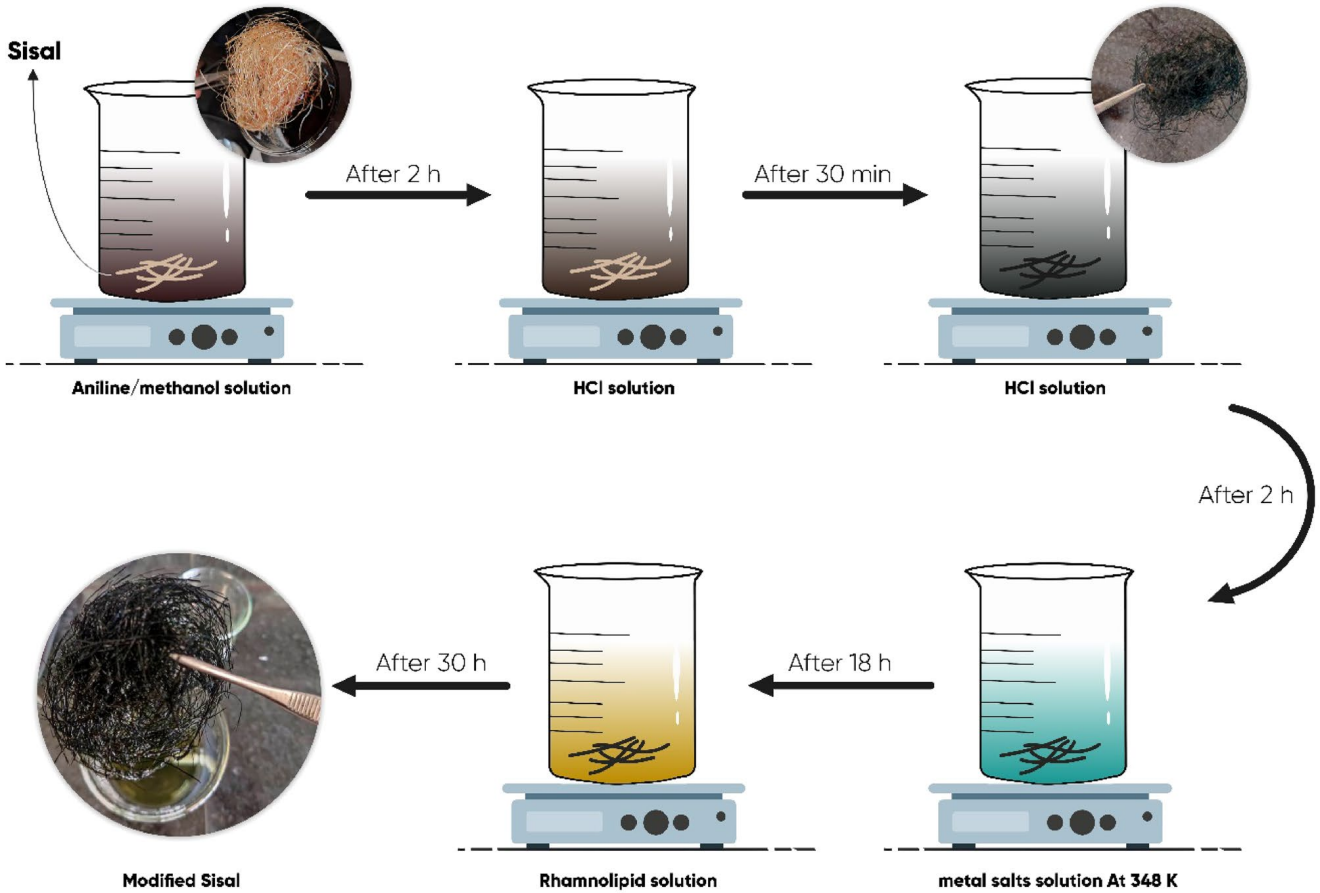
The synthesis procedure of SF@PANI@LDH@RL.

0.610 g of Al(NO_3_)_3_0.9H_2_O and 0.928 g of Ni(NO_3_)_2_0.6H_2_O dissolved in 80 ml water aided by ultrasound for the modification of NiAl-LDH. 0.672 g of the urea and SF@PANI were eventually added to the mixture. This combination reacted at 348 K for 18 h, occurring in the atmosphere condition to acquire binary PANI and NiAl-LDHs composites modified sisal fibers (SF@PANI@LDH). After that, the prepared SF@PANI@LDH was washed entirely and dried at 328 K. As a control experimentation, this urea hydrolysis method modified LDH on the surface of sisal fiber (SF@LDH) directly. A rhamnolipid mixture is made by adding 4 g of rhamnolipid to 50 ml of warm deionized water and stirring gently for 30 min until observing a milky color. Then SF@PANI@LDH was soaked in the rhamnolipid mixture at ambient temperature and pressure for 30 h. At last, the SF@PANI@LDH was washed thoroughly and dried at room temperature. All of the mentioned stages are illustrated in Fig. 1.

## Results and discussion

### Characterization

**Figure 2:**
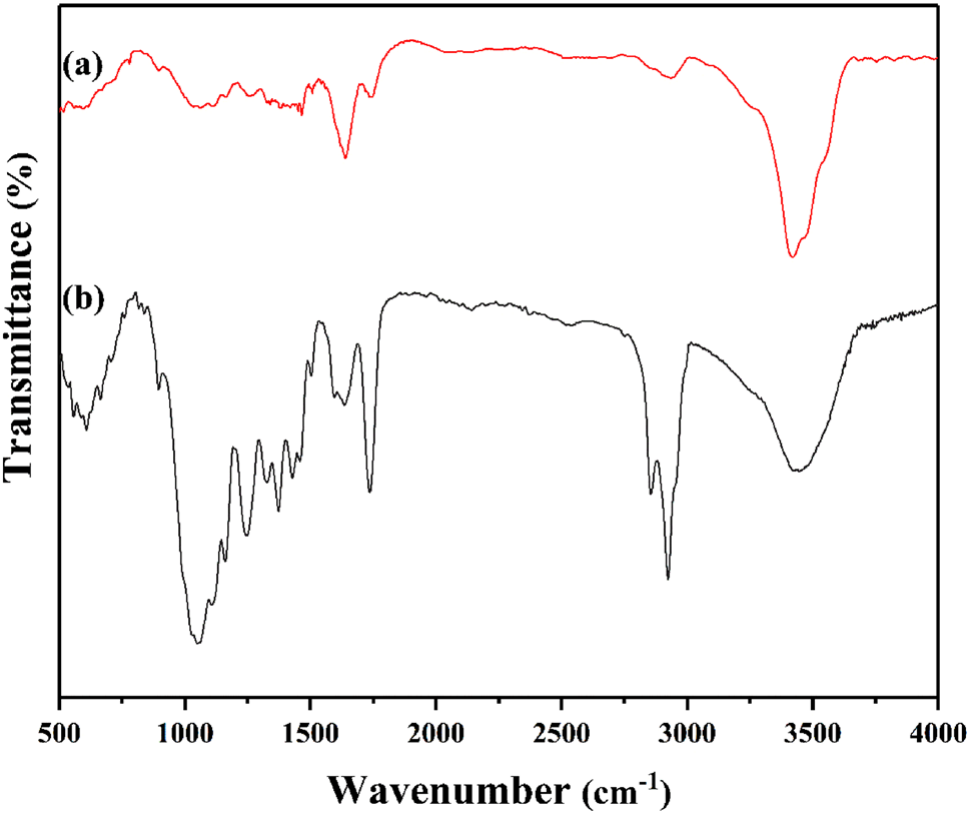
FTIR spectra of raw sisal fiber (**a**), and SF@PANI@LDH@RL (**b**).

Figure 2 displays the FTIR spectra of raw sisal and SF@PANI@LDH@RL. The new bands set at 2928 and 2856 result from C–H stretching vibration appearance. The peaks at 2905 cm^–1^ and 2840 cm^–1^ indicate the C–H stretching vibration of aliphatic hydrocarbons from cotton^34^. Also, the peaks at 1720 cm^–1^ and 1665 cm^–1^ demonstrate the C–O stretching vibration of acetyl groups of lignin and hemicellulose. The peak at 1230 cm^–1^ shows second lignin. Moreover, the new peak at 1739 cm^–1^ expresses the stretching vibrations of C=O groups of rhamnolipid that demonstrates the successful modification. Surface OH of Ni/Al LDH layers and coordinated water stretching vibrations were visible at 3500 cm^–1^ in every component broadband spectra. The peaks observed at 800 cm^–1^ are indicative of the stretching vibrations associated with the metal and oxygen (M–O) bonds present in the samples. The observation of interlayer ${NO}_{3}^{-}$ anions in the LDH was recorded at a wavenumber of 1385 cm^–1^^5^.

**Figure 3:**
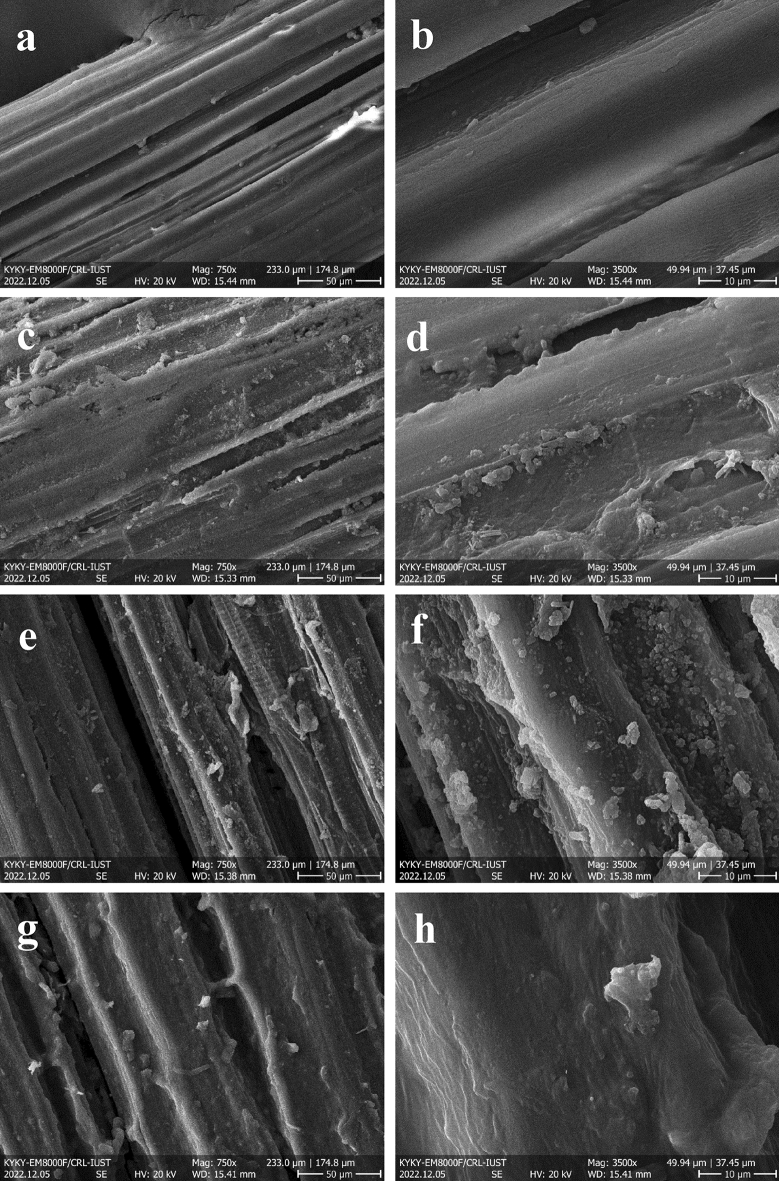
SEM pictures of raw sisal fiber (**a**,**b**), SF@PANI (**c**,**d**), SF@PANI@LDH (**e**,**f**) and SF@PANI@LDH@RL (**g**,**h**).

SEM images of the as-prepared biosorbent are provided in Fig. 3. According to Fig. 3a,b displaying the SEM pictures of plain sisal, which resembles a cylindrical bar with cracks and gaps. It is evident that there is slight porosity on its exterior surface. Figure 3c,d displays sisal after the polyaniline modification. A visual examination of these microstructures indicated that a few spherical particle colonies are created, supplying more porosity and areas valuable in pollutant adsorption. Figure 3e depicts sisal after the transformation with the LDH crystals, a delicate coating of LDH crystals with uniform size and condition developed on the sisal fiber, indicating the successful immobilization of LDHs onto the sisal fiber. The crystals were spread on the surface, delivering adequate functional zones for adsorption. As depicted, before the modification of LDH with rhamnolipid, NiAl-LDH has a thin (lamellar) form and stays in this structure in the company of rhamnolipid. Due to the rhamnolipid shown in Fig. 3f, these layered double hydroxide adsorbents became aggregated. These SEM pictures verify that the synthesized sisal biosorbent might be reassuring for MB and RO16 removal^35–38^.

**Figure 4:**
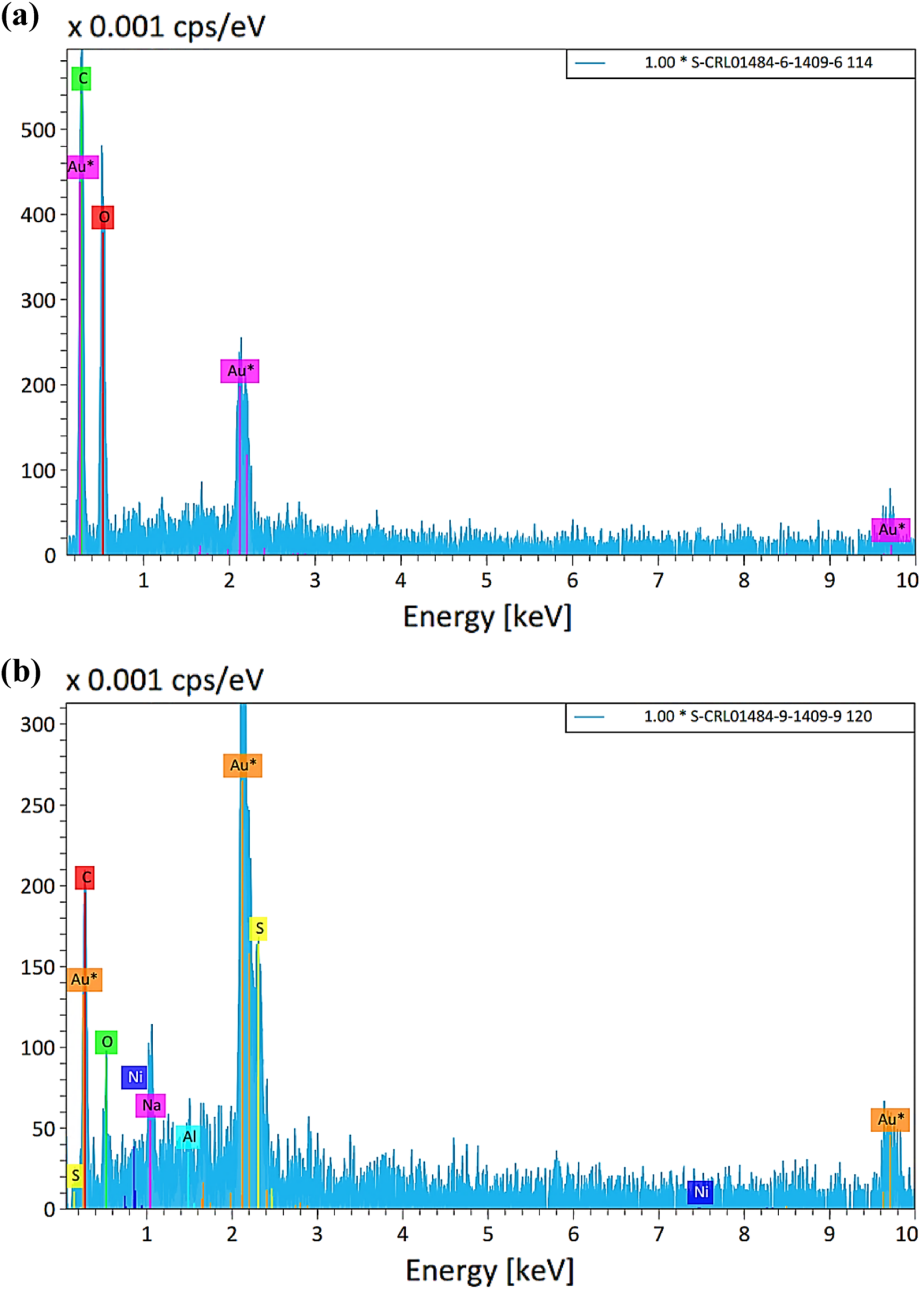
EDS analysis of raw sisal fiber (**a**), and SF@PANI@LDH@RL (**b**).

As shown in Fig. 4, energy-dispersive X-ray analysis was used to analyze the raw sisal fiber and the SF@PANI@LDH@RL element configurations to verify the successful modification. The sample was covered with a thin layer of gold to prevent charging of the surface and deliver a homogeneous surface for examination and imaging. The graph shows the elements of Ni and Al on the surface of SF@PANI@LDH@RL adsorbent. Carbon groups were increased in SF@PANI@LDH@RL after adding rhamnolipid to the adsorbent.

**Figure 5:**
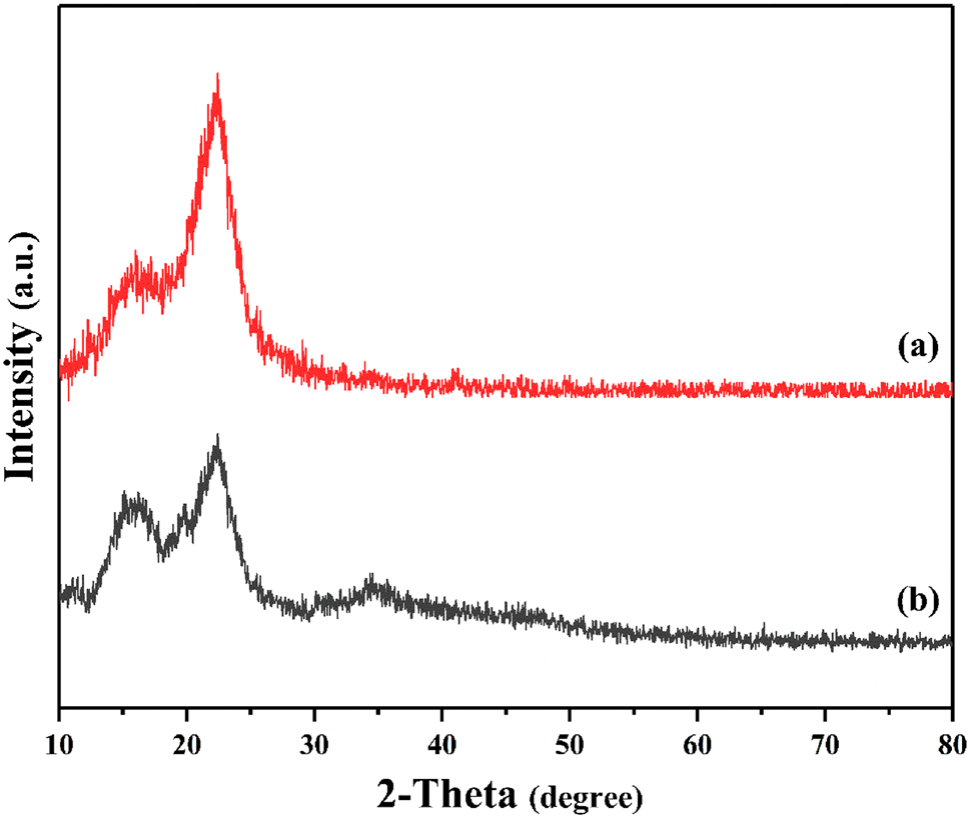
XRD images of raw sisal fiber (**a**), and SF@PANI@LDH@RL (**b**).

Figure 5 shows that the X-ray powder diffraction technique was used to illustrate the finalized samplings of raw sisal and SF@PANI@LDH@RL. The patterns of all materials match the simulated pattern. The graph displays intense peaks at 2 $\theta$ = 15.2°, and 23.9° attributed to the existence of cellulose in sisal fiber. Moreover, peaks at 2 $\theta$ = 11.2°, 35.1° and 39.4° confirmed the successful LDH formation. Employing rhamnolipid will enhance the interlayer distance of the end nanocomposite. According to Fig. 5, XRD patterns confirm that the space between the layers of SF@LDH@RL was more than that of one of the Ni/Al-LDH. This contrast is because of the rhamnolipid anion inside the layers^39–41^.

### Comparison of sisal and SF@PANI@LDH@RL

**Figure 6:**
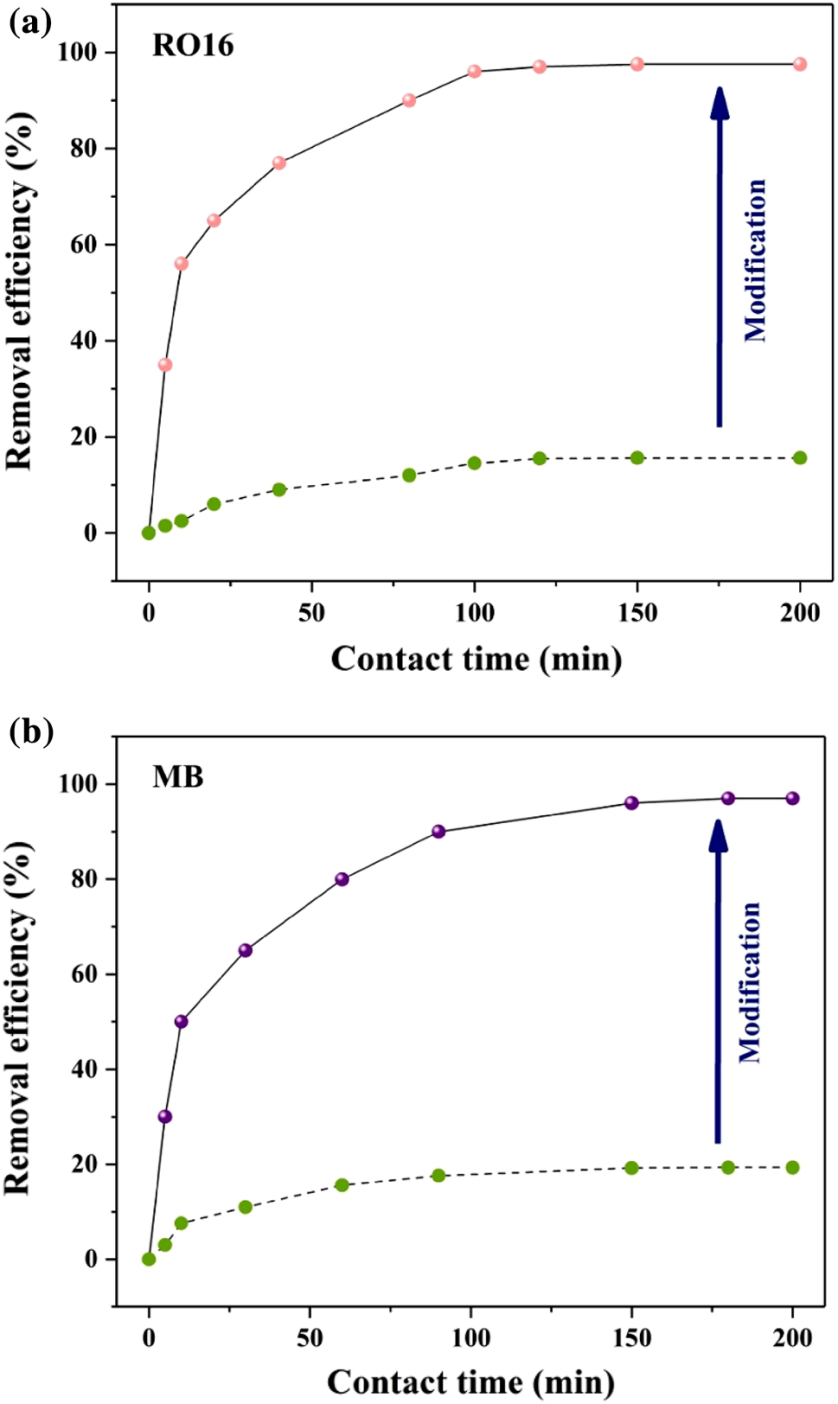
Comparison of SF@PANI@LDH@RL with raw sisal in terms of RO16 removal (**a**) and MB removal (**b**).

Sisal is an economical biosorbent that is a potent fit for dye elimination. Like other natural fabrics, sisal possesses a negative surface that can draw positive varieties with electrostatic attraction assistance. Nonetheless, adsorption will not happen due to repulsive powers when the intended combination has a negative surface. A novel SF@PANI@LDH@RL biosorbent was designed to solve this significant issue. The removal efficiency of RO16 and MB by sisal and SF@PANI@LDH@RL are compared in Fig. 6a and b, respectively. The outcomes demonstrated that the efficiency of SF@PANI@LDH@RL for RO16 was 6.25 and for MB was 5 times greater than raw sisal. Thus, the synthesized SF@PANI@LDH@RL was a potent adsorbent for dye removal.

### The effect of operational factors

#### Effect of contact time

**Figure 7:**
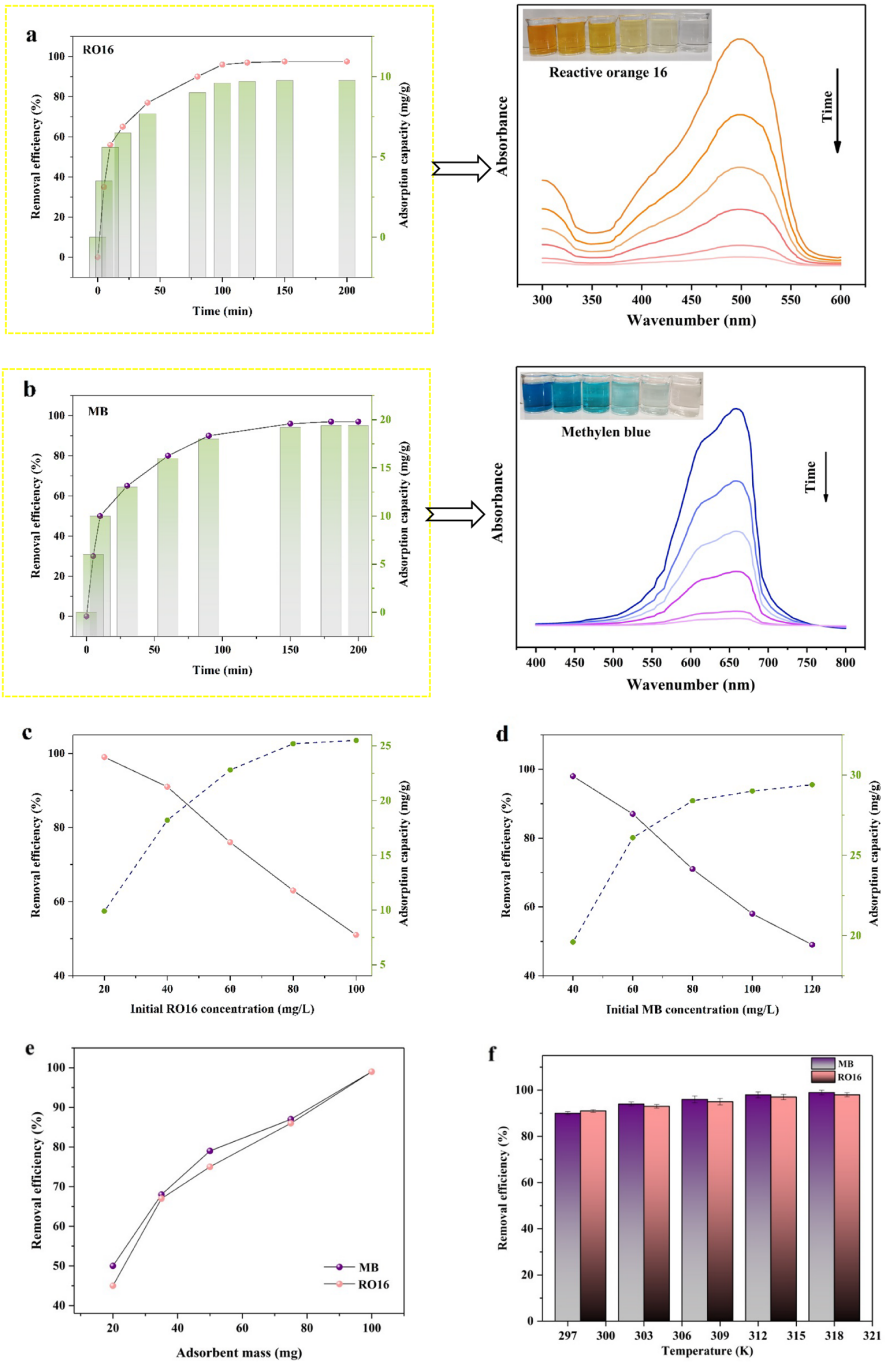
The effect of contact time (**a**,**b**), RO16 concentration (**c**), MB concentration (**d**), adsorbent dosage (**e**) and temperature (**f**).

The influence of contact time enhances the equilibrium adsorption of pollutants between dyes and modified sisal, offering the promising potential for removing MB and RO16. The removal efficiency of RO16 and MB are illustrated in Fig. 7a,b SF@PANI@LDH@RL adsorption capacity for MB and RO16 ranges from 0 to 200 min. According to Fig. 7a,b MB and RO16 uptakes had average speeds and reached equilibrium at 100 min. During this period, more than 90% of MB and RO16 were eliminated from solutions; then, from 100 to 200 min, the adsorption capacity peaked in both graphs. Multiple active areas are initially available for MB and RO16, so the adsorption rate is high before it reaches equilibrium. After reaching the point of equilibrium, the active regions became fully saturated, resulting in the inability of further analyte molecules to be adsorbed into the substances, ultimately leading to the termination of adsorption.

#### Effect of adsorbent dosage

Adsorption dosage is a crucial factor in the adsorption procedure Owing to economic and industrial problems. Utilizing massive quantities of adsorbent raises additional pollution sources in the surroundings and causes a costly purification procedure Fig. 7e shows the MB and RO16 adsorption capacity and removal efficiency results at various adsorbent doses in the range of 20–100 mg. MB and RO16 removal efficiency enhanced from 48 and 20%, respectively, to almost 99% by improving the adsorbent doses from 20 to 100 mg. Accordingly, it is concluded that as the sorbent dose grows, numerous active areas for dye removal become available. A 100 mg sorbent dosage was selected in the subsequent analyses to optimize the remaining aspects because it offered the highest efficiency toward dye elimination.

#### Effect of dye concentration

As displayed in Fig. 7c,d the influence of starting MB and RO16 concentrations on adsorbent performance was also evaluated. The most significant removal efficiency of 99% was detected at both MB and RO16 concentrations of 40 ppm and 20 ppm, respectively. The lowest removal of 40% for MB and 50% for RO16 was seen at 120 mg/L and 100 mg/L, respectively. Thus, all accessible active sites on modified sisal were stable, and the number of pollutant molecules grew as the concentrations of MB and RO16 grew. Higher dye concentrations will result in lower removal efficiency because these required sites have not been accessible and vacant. Nearly all of the MB and RO16 were eliminated. For further experiments, the initial dye concentrations of MB and RO16 were chosen as 40 mg/L and 20 mg/L, respectively.

#### Effect of temperature

The temperature optimization is shown in Fig. 7f. As observed, 313 K has the most acceptable outcome for MB and RO16 and delivers the best adsorption efficiency. It is evident that the two reactions are endothermic because the adsorption is elevated at high temperatures.

#### Effect of pH

The surface charge of the substance and the examined dyes is extensively conditional on the pH of the solution. The pH can adjust the molecular structures of the adsorbates, the surface charge of the adsorbent, and the ionization extent. The pH of the solution plays a crucial role in determining the interaction between the adsorbent and the dyes MB and RO16, as it governs the ionization behavior of the species present in the solution^42^.

**Figure 8:**
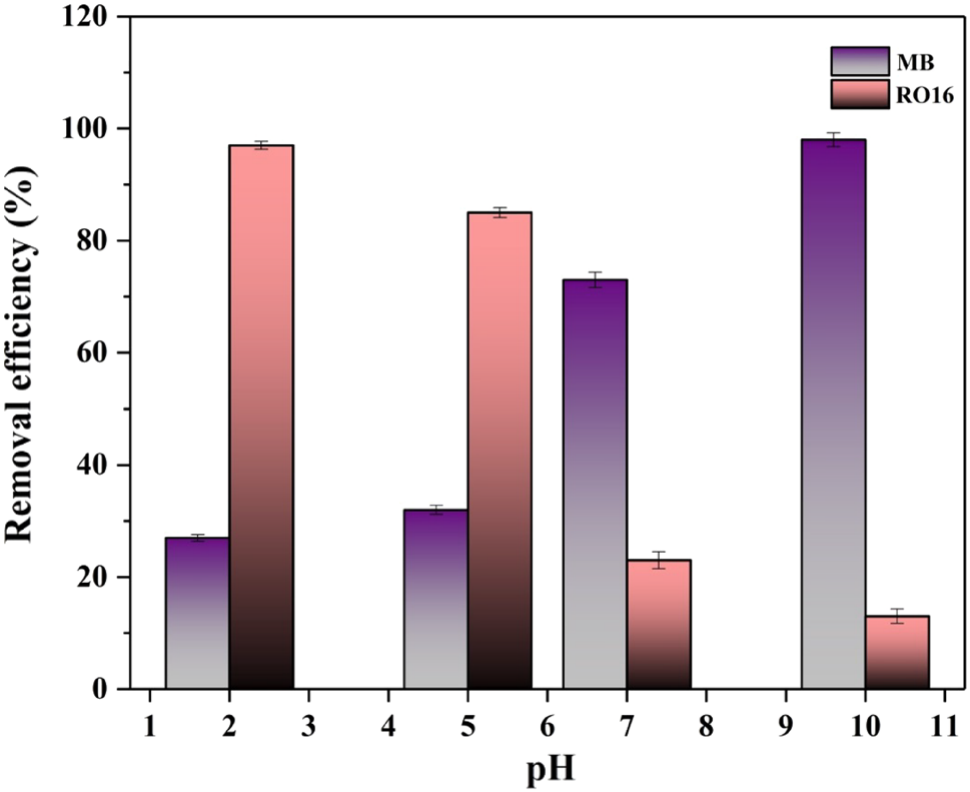
The effect of pH for RO16 and MB removal by SF@PANI@LDH@RL.

As depicted in Fig. 8, the adsorption efficacy of MB is observed to decrease upon lowering the pH to the acidic range. According to the graph, the best pH for MB elimination is 10, which will be utilized for further investigations. MB has π-conjugated electron density in its construction, but the suggested SF@PANI@LDH@RL does not possess π-conjugated electrons; consequently, it will exclude π-π stacking interaction. Simultaneously, the adsorbent may interact with positively charged MB through cation-π interaction, occurring effectively on both the upper and lower sides of the MB. Furthermore, the modified sisal can form hydrogen bonds with the nitrogen zone of the dye via its hydroxyl groups, leading to efficient adsorption. In contrast, the graph depicts a complete reversal for the RO16. The molecular structure in question lacks an extended π-conjugated arrangement. The adsorption efficiency of RO16 reached its apex by reducing pH to 2. According to the graph, the best pH for RO16 elimination is 2, which will be utilized for further investigations. RO16 ionizes into anion formation at low pH, influencing the electrostatic interaction with the adsorbent with a positive charge. Furthermore, the existence of hydroxyl groups in the RO16 molecule results in the formation of hydrogen bonds with the hydroxyl functional groups present on the proposed adsorbent.

### Adsorption isotherms

Understanding the partitioning behavior of MB and RO16 molecules with the solution and SF@PANI@LDH@RL as an adsorbent is essential to design an optimum adsorption method. This article will present several commonly used adsorption isotherm equations, such as Temkin, Langmuir, and Freundlich, in order to analyze experimental data obtained from the adsorption process^43^. First of all, the equation below demonstrates the non-linear form of the Langmuir isotherm:

$${q}_{e}=\frac{{q}_{m}{K}_{L}{C}_{e}}{1+{K}_{L}{C}_{e}}.$$

${q}_{e}$ (mg/g) shows the equilibrium adsorption capacity, and ${q}_{m}$ (mg/g) represents the maximum adsorption capacity. ${K}_{L}$ (L/mg) indicates the constant of the model, and ${C}_{e}$(mg/L) shows the equilibrium concentration of dye. The basics of Langmuir isotherm are presented as a dimensionless equilibrium parameter (${R}_{L}$):

$${R}_{L}=\frac{1}{1+{K}_{L}{C}_{0}}.$$

${C}_{0}$ (mg/L) is the maximum initial concentration of the analyte, and ${K}_{L}$ is the Langmuir constant. ${R}_{L}$ shows the variety of adsorption to be either favorable (0 < ${R}_{L}$< 1), unfavorable (${R}_{L}$> 1), linear (${R}_{L}$= 1), or irreversible (${R}_{L}$= 0). Langmuir isotherm considers homogeneous adsorption and single-layer coverage of the modified sisal surface by dye without any interaction among analyte molecules. Hence, Freundlich isotherm was proposed as a reversible model, which is a model for surface heterogeneity and not restricted to monolayer formation to fix the mentioned problem:

$${q}_{e}={K}_{f}{C}_{e}^{1/n}.$$

${q}_{e}$ (mg/g) shows the equilibrium adsorption capacity, and ${C}_{e}$(mg/L) represents the equilibrium concentration of dye. ${K}_{F}$ (mg/L) is the relative adsorption capacity, and $n$ indicates adsorption intensity. The adsorption heat among MB and RO16 and modified sisal is defined by the Temkin model, which is presented as the following equation:

$${q}_{e}=\left(\frac{RT}{{b}_{T}}\right)\mathrm{ln}\left({K}_{T}{C}_{e}\right).$$

**Figure 9:**
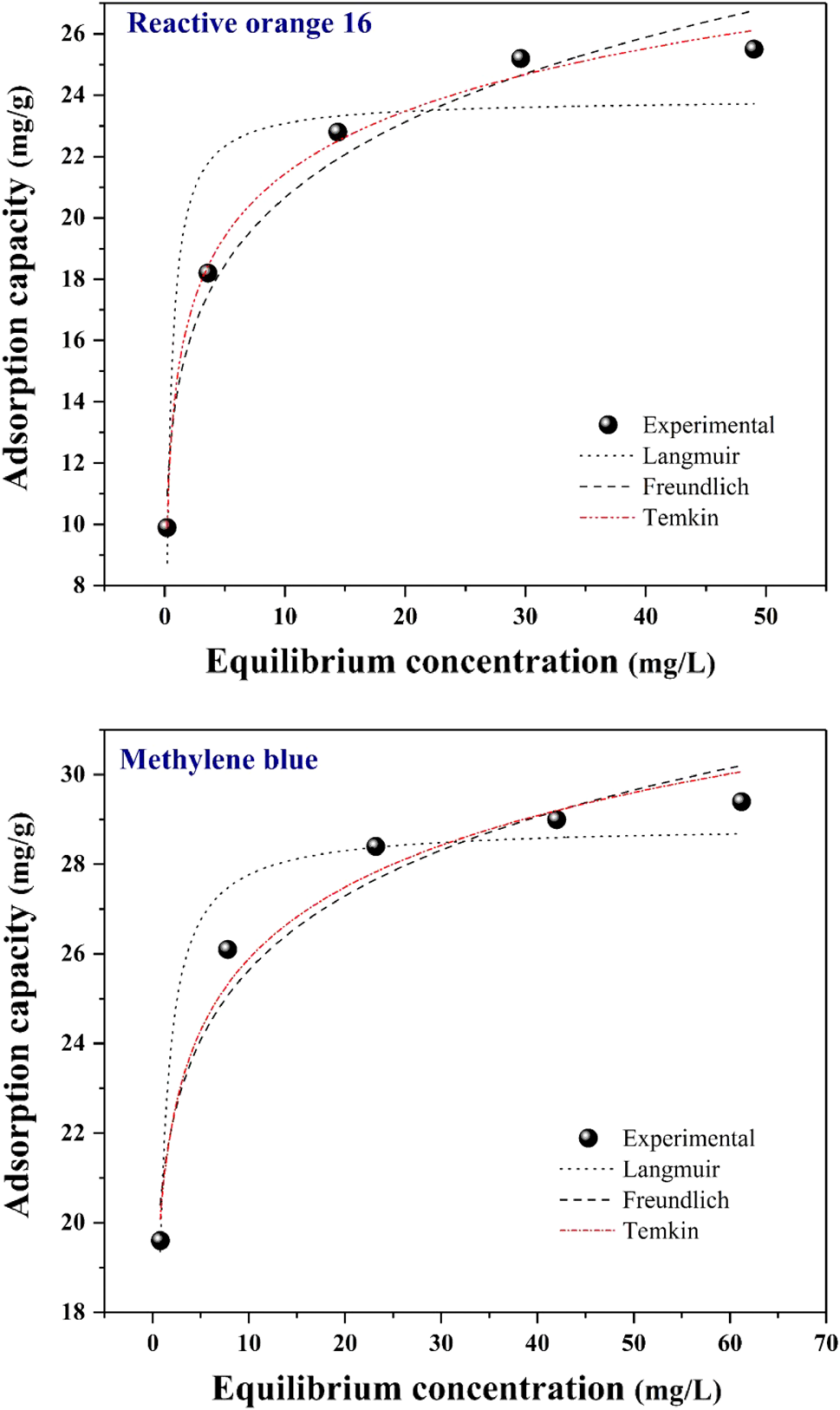
Isotherm fitted curves (non-linear) of RO16 and MB adsorption.

$R$ represents the universal gas constant (8.314 J/mol. K), ${b}_{T}$ (J/mol) has a relation with the adsorption heat constant, ${K}_{T}$ shows the equilibrium binding constant (L/g), and $T$ (K) is the temperature. The evaluation of the adsorption isotherm of RO16 and MB for modified sisal was conducted at a temperature of 298 K. The statistical analysis was performed using the Temkin, Langmuir, and Freundlich isotherms. Figure 9 depicts the relationship between the adsorption capacity and the equilibrium concentrations of MB and RO16, utilizing a non-linear methodology. In both cases of MB and RO16 elimination, the ${R}^{2}$ values of the Temkin isotherm are higher (0.983 for MB and 0.996 for RO16), which concurs with experimental adsorption statistics. The Temkin isotherm model deems the binding energies spread uniformly and considers the indirect reaction between the modified sisal and the dyes. The Temkin isotherm hypothesis is that the adsorption heat declines linearly when the surface coverage grows. The outcomes proved that the adsorption procedure of MB and RO16 could be regarded as a chemisorption procedure. Temkin isotherm allows measuring the heat of the adsorption; positive ${b}_{T}$ will result in an exothermic process. Furthermore, other ${R}^{2}$ values are not small. Regarding MB removal, the ${R}^{2}$ value for the Freundlich model was 0.965 and for the Langmuir model was 0.959. Regarding RO16 removal, the ${R}^{2}$ value for the Freundlich model was 0.981 and for the Langmuir model was 0.914. Based on the Temkin model, the *n* factor makes significant values regarding the adsorption procedure. $\frac{1}{n}<1$ according to our estimation; therefore, chemical bonds can be formed on the heterogeneous surface of modified sisal in dye elimination. Consequently, the heat adsorption of all dyes among the layers declined coverage of the adsorbent nanocomposite^13,31^. Table 1 shows numerical studies of isotherm models.

**Table 1:** Adsorption isotherm models for RO16 and MB adsorption on the SF@PANI@LDH@RL.

Isotherm models	Important parameters	Reactive orange 16	Methylene blue
Langmuir	${q}_{m}$*(mg/g)*	23.879	28.859
	${K}_{L}$*(L/g)*	2.89	2.545
	${R}^{2}$	0.914	0.959
Freundlich	${K}_{F}$*(mg/L)*	14.206	20.816
	$n$	6.148	11.054
	${R}^{2}$	0.981	0.965
Temkin	${b}_{T}(J/mol)$	883.105	1130.082
	${K}_{T}$*(L/mg)*	144.328	7652.461
	${R}^{2}$	0.996	0.983

### Adsorption kinetics

**Figure 10:**
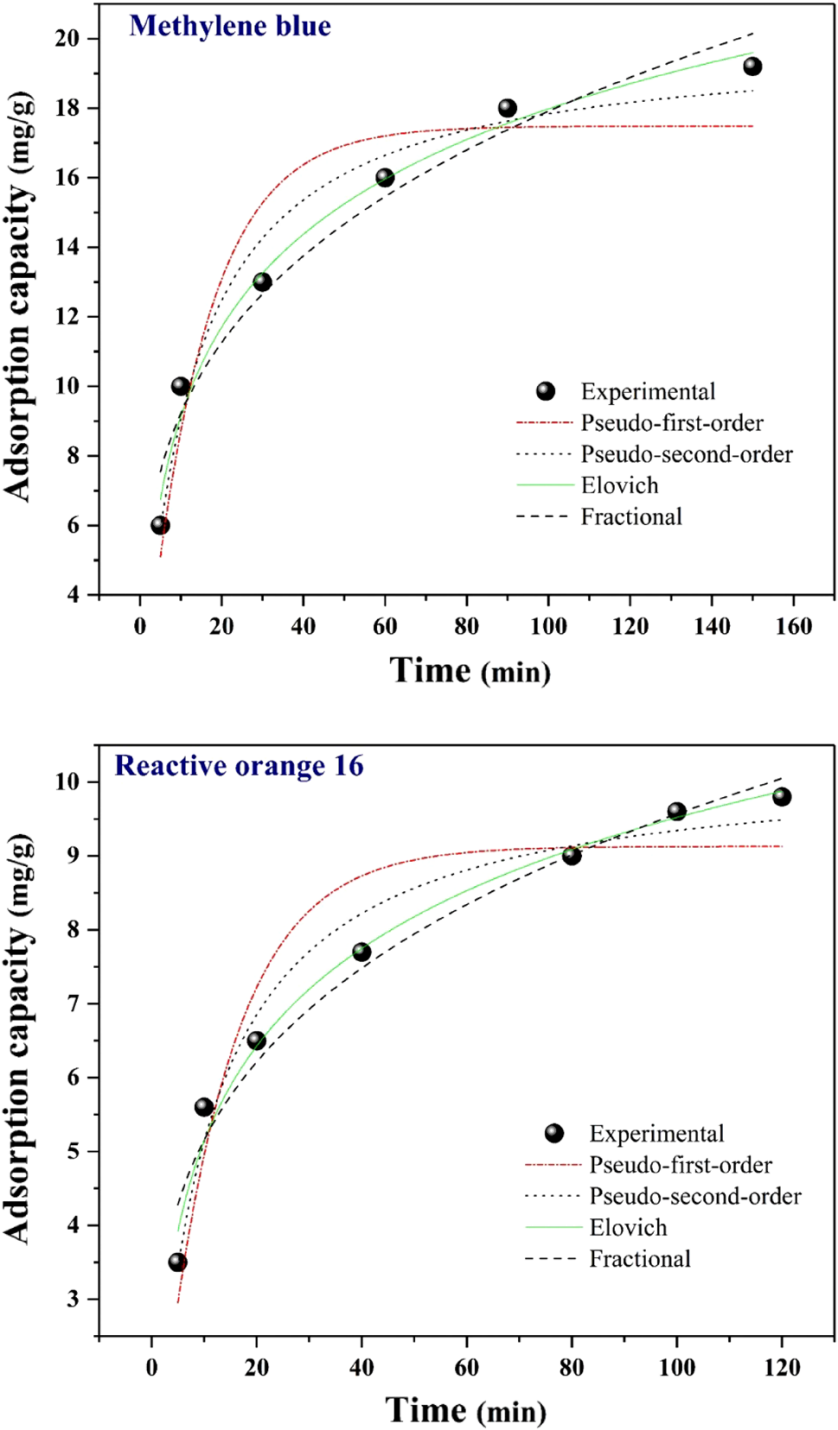
Kinetic plots of MB and RO16 adsorption.

Kinetic factors mostly describe the adsorption efficiency since rapid kinetics is vital in aqueous medium adsorption. Four standard kinetic models, including pseudo-first-order (PFO), pseudo-second-order (PSO), Elovich, and fractional kinetic models, were provided for the kinetic factors of modified sisal to evaluate their experimental setup. The outcomes achieved from the kinetic models and the non-linear curve fittings are shown in Fig. 10 and Table 2 for MB and RO16. The pseudo-first-order kinetic model is represented in the below equation:

**Table 2:** Kinetic parameters pertaining to the adsorption of MB and RO16 on SF@PANI@LDH@RL.

Kinetic model	Parameters	Reactive orange 16	Methylene blue
PFO	${q}_{e}$(mg/g)	9.131	17.482
	${K}_{1}$(1/min)	0.078	0.067
	${R}^{2}$	0.911	0.913
PSO	${q}_{e}$(mg/g)	10.282	19.991
	${K}_{2}$(g/mg min)	0.009	0.004
	${R}^{2}$	0.976	0.973
Elovich	$\alpha$	2.504	3.505
	$\beta$	0.511	0.249
	${R}^{2}$	0.987	0.986
Fractional	${K}_{p}$	2.781	4.742
	${V}_{p}$	0.268	0.288
	${R}^{2}$	0.972	0.964

$${q}_{t}={q}_{e}\left(1-{e}^{-{k}_{1}t}\right),$$

$$\mathrm{ln}({q}_{e}-{q}_{t})=\mathrm{ln}{q}_{e}-{k}_{1}t.$$

The equation below represents the PSO model, which defines the adsorption kinetics:

$${q}_{t}=\frac{{k}_{2}{q}_{e}^{2}t}{1+{k}_{2}{q}_{e}t},$$

$$\frac{t}{{q}_{t}}=\frac{1}{{k}_{2}{q}_{e}^{2}}+\frac{t}{{q}_{e}}.$$

${k}_{1}$ (1/min) and ${k}_{2}$ (g/mg.min) are the rates constant of the PFO and PSO models, respectively.

Temkin adsorption isotherm equation is the root of the Elovich kinetic model. Elovich model is employed chiefly to define the chemical adsorption procedure of gas on a solid surface. The following equation represents the Elovich kinetic model:

$${q}_{t}=\frac{1}{\beta }\mathrm{ln}\left(\alpha \beta t+1\right).$$

The correlation coefficient values (${R}^{2}$) were compared to determine the suitable kinetic model. As demonstrated in Table 2. (0.987, 0.986 for RO16 and MB dyes, respectively) found by Elovich were more significant than others. Accordingly, Elovich is the best-fitting model for both dyes and proves that external mass transfer and chemisorption rate could manage the dye adsorption procedures. Moreover, ${R}^{2}$ values obtained from Elovich for RO16 and MB were closer to one than the other models, indicating the suitability of Elovich to declare the adsorption kinetic. $\alpha$ represents the desorption constant (g/mg), equal to the first adsorption rate (mg/g.min), and is a constant that relates to the activation energy for chemisorption and the surface coverage. These outcomes indicate that numerous systems are involved in the adsorption of MB and RO16 by modified sisal. The equation below represents the non-linear form of the fractional power:

$${q}_{t}={k}_{p}{t}^{{V}_{p}}.$$

The antilogarithm of the intercept refers to the ${k}_{p}$ value. ${q}_{t}$ is the amount of adsorbed analyte at t (min). ${V}_{p}$ is a constant typically lower than one if adsorption kinetic statistics fit into the power function model. As displayed in Table 2, ${R}^{2}$ values of the Fractional power kinetic model were 0.972 for RO16 and 0.964 for MB. Compared with other models, the fractional power kinetic model cannot sufficiently define the experimental statistics^44–46^.

### Adsorption thermodynamics

Table 3 displays Vant’s Hoff thermodynamic parameters for MB and RO16 dyes. The equations below express the calculation of Thermodynamic parameters (ΔG°, ΔS°, ΔH°):

**Table 3:** Thermodynamic parameters for MB and RO16 adsorption onto the SF@PANI@LDH@RL.

Dye	$\Delta \mathrm{H}^\circ$ (J/mol)	$\Delta \mathrm{S}^\circ$ (J/mol.K)	$\Delta \mathrm{G}^\circ$ (J/mol)				
			298 K	303 K	308 K	313 K	318 K
RO16	59,495.81	270.01	− 20,968.05	− 22,318.12	− 23,668.18	− 25,018.25	− 26,368.31
MB	85,368.15	356.18	− 20,773.48	− 22,554.38	− 24,335.28	− 26,116.18	− 27,897.08

$$\Delta \mathrm{H}^\circ -\mathrm{T}\Delta \mathrm{S}^\circ =-{\mathrm{RTlnK}}_{\mathrm{D}},$$

$$\Delta \mathrm{G}^\circ =\Delta \mathrm{H}^\circ -\mathrm{T}\Delta \mathrm{S}^\circ ,$$

$$\Delta \mathrm{G}^\circ =-{\mathrm{RTlnK}}_{\mathrm{D}},$$

R represents the universal gas constant [8.314 J/(mol·K)], and T refers to temperature (K). ${\mathrm{K}}_{\mathrm{D}}$ indicates the distribution coefficient of the adsorption. Based on Table 3, the ΔH° values were positive in both dyes, demonstrating an endothermic approach in which more elevated temperatures lead to greater sorption for RO16 and MB over modified sisal. Typically, enthalpy values of more than 40 kJ/mol define chemisorption. In this work, enthalpy was 70.98 kJ/mol for RO16 and 84.10 kJ/mol for MB, showing a chemical adsorption procedure of dye molecular substances onto modified sisal. Table 3 shows that higher temperatures lead to more negative values of ΔGº in both RO16 and MB, and negative ΔG° values demonstrate that the adsorption process is spontaneous. So the approach was more spontaneous and possible at higher temperatures. Intercept allows understanding $\Delta \mathrm{S}^\circ$ (kJ/mol.K). Positive values of $\Delta \mathrm{S}^\circ$ in both RO16 and MB demonstrate that the system becomes more disordered. These outcomes are in sync with the experimental works of temperature impact and Temkin conclusions^18,47^.

### Adsorption mechanism

**Figure 11:**
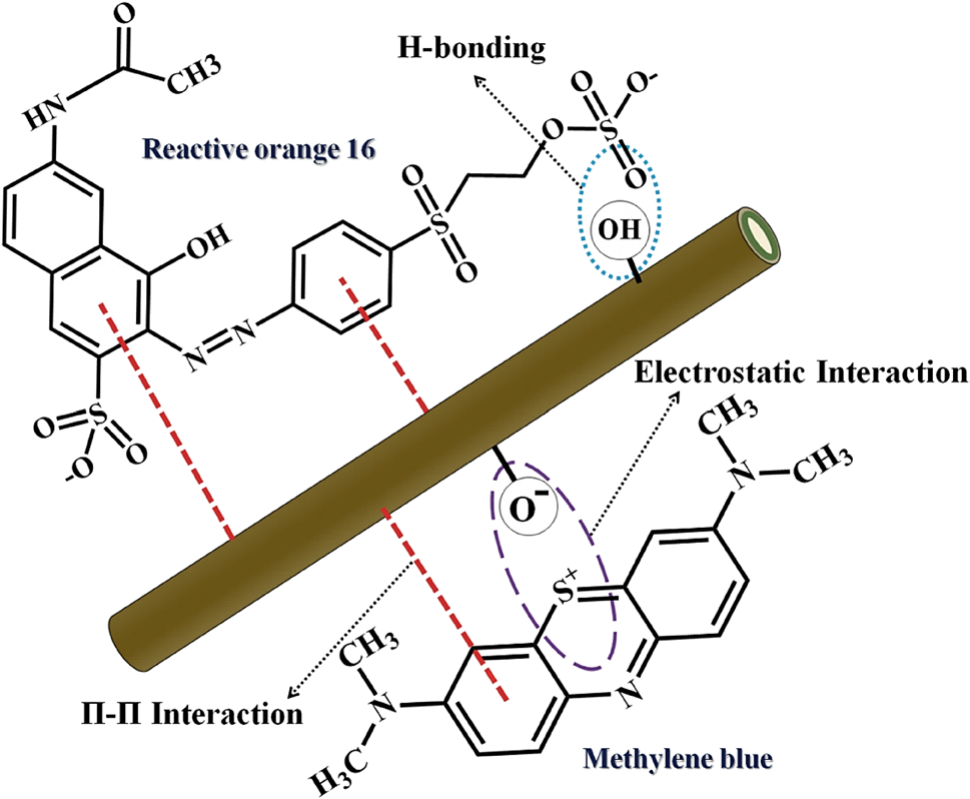
Dye removal mechanism by SF@PANI@LDH@RL.

Typically, hydrogen bonds, van der Waals energies, electrostatic attraction, π–π stacking, base-acid reactions, and hydrophobic contact are the primary tools applied to water pollutants adsorption on adsorbents. In this work, substituting analytes and dyes with the interlayer anion of SF@PANI@LDH@RL is the primary cause and motivator of the adsorption approach. Different interactions simplify the adsorption occurrence. Regarding MB elimination, an interaction can occur between the π-conjugated electrons and SF@PANI@LDH@RL as a π-cation interaction with a positive charge. Because of the basic pH of the adsorption procedure, moderate deprotonation of the LDH can lead to electrostatic interaction with MB, which facilitates the adsorption. In relation to the elimination of RO16, it can be observed that the moderately protonated state of RO16 was a result of the acidic pH of adsorption. This, in turn, hindered the electrostatic attraction with SF@PANI@LDH@RL. The presence of electron density associated with π orbitals may give rise to π-cation interactions between RO16 and SF@PANI@LDH@RL. In addition, the hydroxyl (OH) functional groups have the ability to form hydrogen bonds with the hydroxides present in SF@PANI@LDH@RL. Figure 11 illustrates the removal mechanism of dyes by SF@PANI@LDH@RL.

### Reusability of SF@PANI@LDH@RL

**Figure 12:**
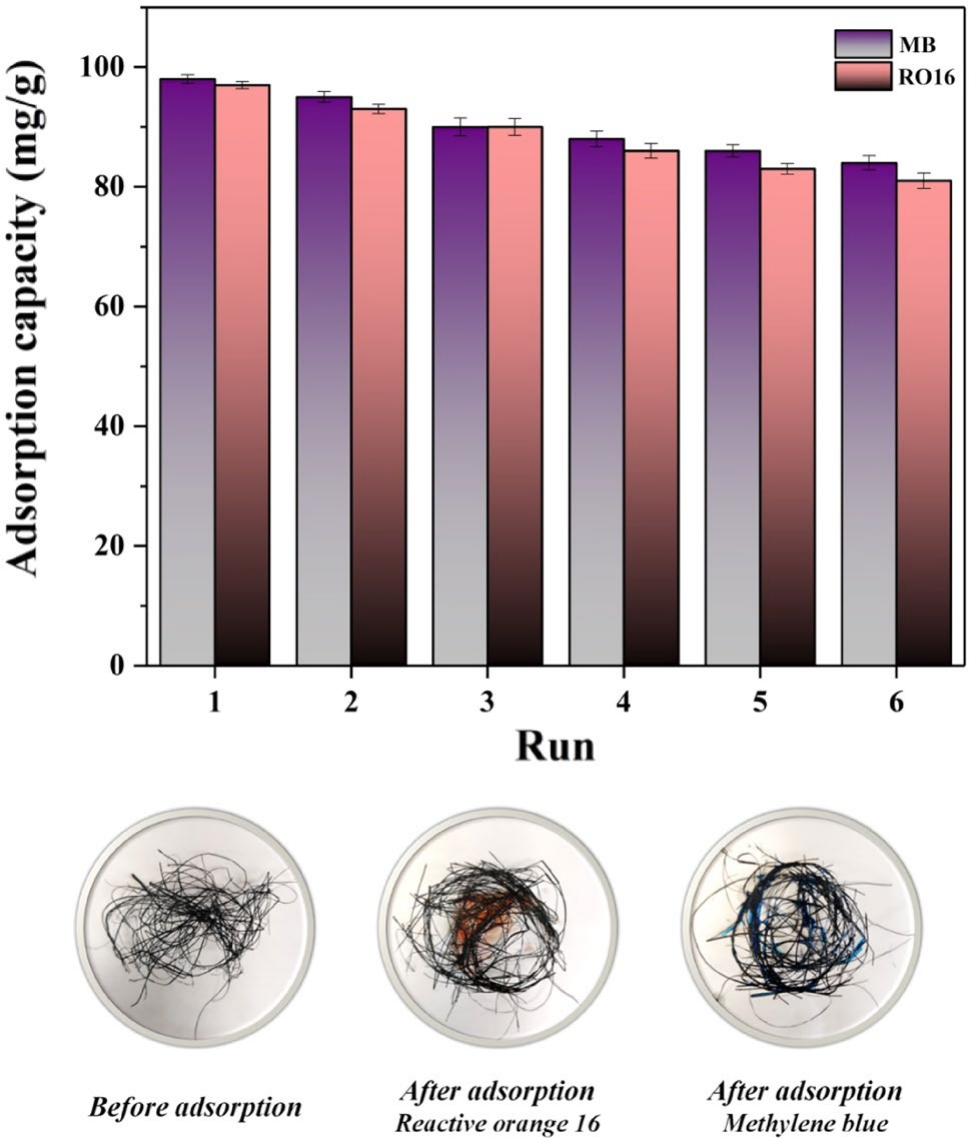
Reusability of SF@PANI@LDH@RL for RO16 and MB adsorption.

The reusability of the adsorbent has a crucial role in adsorption experiments on lab and industrial scales. This work investigated the reusability of SF@PANI@LDH@RL under optimum conditions, and Fig. 12 displays the outcomes. After washing the used sorbent with distilled water and ethanol and drying it at room temperature, SF@PANI@LDH@RL adsorbent was reused six consecutive times. Nevertheless, the adsorption efficiency was still higher than 80% for MB and RO16 even after utilizing SF@PANI@LDH@RL adsorbent six times. Thus, the outcomes indicated that SF@PANI@LDH@RL offers promising reusability in repetitious series to remove dye from aqueous solutions.

### Comparison with other adsorbates

SF@PANI@LDH@RL adsorbent was compared with earlier reports in the following table, emphasizing the benefits of current results concerning adsorption capacity. All results and items are shown in Table 4. As shown, SF@PANI@LDH@RL adsorbent successfully removes MB and RO16 from aqueous solutions and has a superior adsorption capacity compared to previous reports.

**Table 4:** Comparison of various adsorbents for MB and RO16 removal.

Adsorbent	Adsorbate	Adsorption capacity (mg/g)	Ref
Fe-BDC MOF	MB	8.65	^48^
Mesoporous silica nanoparticles	MB	19.26	^49^
g-C_3_N_4_ loading on NiCo LDH	MB	25.16	^50^
CNT/eucalyptus derived activated carbon-based	MB	49.61	^51^
MR-Co/Al LDH	MB	54.01	^52^
Zeolite/activated carbon@MnO_2_ composite	MB	67.56	^53^
Sep-AAM	MB	99.92	^54^
SF@PANI@LDH@RL	MB	24.813	Present study
AC@nZVI/Ni	RO16	10.53	^55^
Modified zeolites	RO16	12.6	^56^
Activated carbon prepared from rice husk ash	RO16	13.32	^57^
MR-Co/Al LDH	RO16	53.04	^52^
Arachis hypogaea pod powder	RO16	56.48	^58^
*m*-Cs-PVA/FA	RO16	123.8	^15^
SF@PANI@LDH@RL	RO16	23.981	Present study

## Conclusion

Eliminating organic dyes has become a global problem due to different human disorders. The adsorbent in its original state was utilized for the purpose of eliminating MB and RO16, resulting in an adsorption capacity of 24.813 and 23.981 mg/g, respectively. The optimization of the adsorption process involved the identification and manipulation of key factors such as contact time, pH, and adsorbent dosage. Moreover, the modeling analyses were conducted to gain a deeper understanding of the adsorption mechanisms. As mentioned, the Temkin isotherm was the best fit for both dye adsorption investigations showing the monolayer adsorption procedure. Moreover, the Elovich model delivered the most suitable model of the adsorption kinetics of RO16 and MB. At last, Low cost, high removal efficiency, and environmentally friendly are the distinctive benefits of SF@PANI@LDH@RL, which will supply a facile platform for dye removal from effluent wastewater. In forthcoming investigations, SF@PANI@LDH@RL could also be utilized for different impurities, including pharmaceuticals and pesticides.

## Data Availability

All data generated or analyzed data for the experimental part of this study are included in this published article. The data that support the findings of this study are available from the corresponding author, [Mehrdad Negarestani], upon reasonable request. Moreover, all other data that support the plots within this paper and other findings of this study are available from the corresponding author upon reasonable request.
